# Evolution of the Danube River’s islands on the Bulgaria-Romania border over the last century

**DOI:** 10.1371/journal.pone.0317711

**Published:** 2025-01-30

**Authors:** Gabriela Ioana-Toroimac, Marina Vîrghileanu

**Affiliations:** 1 Faculty of Geography, University of Bucharest, Bucharest, Romania; 2 Francisc I. Rainer Institute of Anthropology, Romanian Academy, Bucharest, Romania; Fisheries College and Research Institute, INDIA

## Abstract

The border line between Bulgaria and Romania was established a century ago on the Danube River’s thalweg, going around islands. Over time, islands migrated; to avoid conflicts, islands located on the border line are declared neutral territory by both countries and they exit from use. In this context, the paper aims to draw conclusions on the spatial dynamics of the Danube River’s sandy islands along the Bulgaria-Romania border. This approach is mostly a GIS work on various documents over the last century, with interpretations specific to fluvial geomorphology. Our paper showed patterns of evolution of islands. (i) The trajectory of half of the islands can be reconstituted over the last century (i.e., merging with each other and/or migrated). The trajectory of the other half of the islands can be redrawn over the last half of the century. The form and dimensions of islands did not statistically change at any time scale. (ii) At the scale of the last decades, we noticed the slow formation of bars by lee deposition due to sediment excess and then migration of alluvial bars until merging with an island or with the bank, followed by stabilization due to vegetation recruitment. (iii) Islands and their bars migrated on the countries’ border line, but their evolution is slow in time. Our paper invites national authorities to periodically inventory the geomorphological trajectory of islands and bars, but to transfer the decision-making process for stable islands at local scale in order to put them into green use with benefits for local communities.

## Introduction

Fluvial islands are the dominant geomorphic expression of multichannel rivers [1] with impact on the flow structure and morphology [2]. Islands are emerged landforms within a river channel that is separated from the floodplain by water on all sides [3]. Islands are generally not overtopped at bankfull stage whereas a bar may be submerged [1]. Islands are the result of the interplay between flows, sediments and woody vegetation [4,5]. Vegetation is generally a good indicator of stability [4]. Stable alluvial islands are specific to the anabranching pattern [6]. Islands can be indicators of the general health and energy of the hydrosystem [7]. Yet, the presence of islands can also impact the forms and processes within the river channel [1,2]. So far, limited attention has been given to the evolution of fluvial islands whose shape and size change with river processes [8]. A classification system of fluvial islands was developed by J.R. Wyrick and P.C. Klingeman [9] based on the findings of W.R. Osterkamp [10]. The authors aimed to unite features reflecting island formation and river processes that are useful for islands management as part of the hydrosystem. The classification matrix provides connections between the process mechanisms (island types) and the channel responses (island characteristics). Each channel type has specific islands. Islands can form through avulsion, gradual degradation of the channel, lateral shifts, bar/riffle stabilization, structural, flood deposits, lee deposition, mass movement, and damming. Depending on their origins, the fluvial islands can have certain features in terms of geometry, land cover, and dynamics.

The fluvial islands are integrated parts of the hydrosystem and more precisely of the river corridor. Therefore, the islands follow the trajectory of the river evolution, depending, in general, on the climate and human pressures [11]. As examples, the majority of the large rivers of Europe lost their islands over the period of major human interference [4]. The Rhone in France, the Rhine in Germany, the Waal in the Netherlands, the Danube in Austria, and so on, are examples of European rivers characterized, before 1900, by reaches with multiple channels and wooded islands [4]. The 19^th^ century anastomosing channel of the Drava River in Hungary-Croatia became straightened and nowadays, small islands develop behind the groins [12]. The Loire in France maintained its islands, but they become colonized by vegetation [13]. Along the Neris River in the North-Eastern Europe, the area of fluvial islands increased due to the reduction of the flood plain area and shorter duration of floods [14]. The Mississippi in the United Stated of America is still creating new islands by trapping sediments in lack of specific maintenance works [15]. Under global warming, the Lena River is creating new islands from sediments available due to the permafrost melting [16].

Studying and understanding the fluvial islands is especially important on countries border. Rivers are commonly used to define political borders [17]. Yet, what is obvious for the geographers, it has always been challenged by the historians, politicians, ideologists etc. [18,19]. Historically, rivers were chosen for boundaries between countries, because of their potential defensive capability, easiness to control, and their visibility on the ground [20]. Yet, rivers have dynamic natural features and processes, such as erosion and sedimentation–gradual or brutal during floods–that can complicate the definition of the boundary. Fluvial dynamics can shape the river borders as shown in several case studies with implication on land property, the access to water resources, and navigation, triggering political conflicts [21–26]. Management strategies can shape differently the two river banks [27] and generally, the development of border areas [28,29].

While the rivers are expected to be messy [30], the borders should be stable to build institutional trust [31]. The border line between Bulgaria and Romania on the Danube River dates back to a convention in 1908 [32]. The border between the two states followed the thalweg corresponding to the lowest depths of the river. The convention stipulated the study of the thalweg by both neighbor countries in order to be able to establish to whom belong the islands appearing on the Danube function of the border line [32]. Nowadays, in practice, a Bulgarian-Romanian Commission is meeting every year and establishes islands that have a neutral character due to their position on the border line or in the close vicinity. The neutral islands have property and access issues with several social and economic implications: they cannot be surveyed and mapped; therefore, they cannot be part of the spatial development plans and strategies or integrated in economic activities.

In this contradictory context, dynamic natural environment versus necessity for stable borders, it is mandatory to better understand the fluvial geomorphology of the Danube River in order to inform the border management. The aim of this paper is to understand the formation and spatial dynamics of the Danube River’s islands since establishing the Bulgaria-Romania border line. How an island is formed and what happens with it over time? The study relies on physico-geographical indicators estimated mostly on maps and satellite imagery, with validation by field observations at local scale. Our study represents a comprehensive analysis of fluvial dynamics at regional scale and contributes to better understanding the anabranching hydrosystems with low energy. In secondary, it raises the question of countries border line and area management.

### Study area

**Fig 1:**
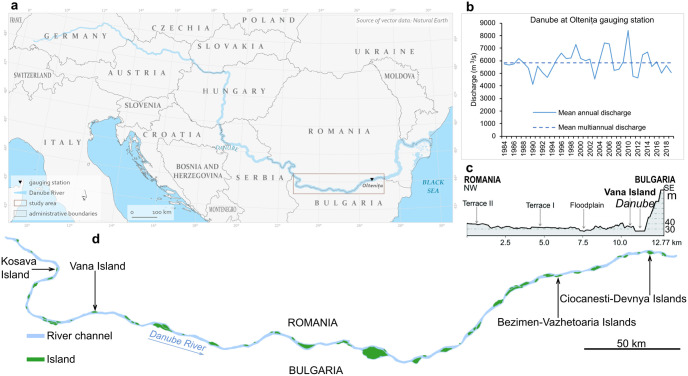
The study area: a) the geographical position in Europe (original map based on free vectors from Natural Earth Data); b) the recent hydrological variability of the Danube (redrawn from data of [37]); c) topographic profile on Danube River banks in the vicinity of Vana Island (redrawn from [38]); d) the geographical position of islands used as demonstrative case studies in this paper (original map based on vectorized data (2016–2022)–according to the methodology section).

The Danube springs in Germany (from the Black Forest Mts.) and exits in Romania/Ukraine (into the Black Sea through a delta). Along 2,857 km, the Danube crosses 4 capitals, 10 countries and collects the waters of 19 countries. The Danube is the natural link between the West and the East inside Europe, connecting different geographical, economic, political regions with various ethnic, religious, historical background, and the countries with the seas [33]. The Danube is a symbol of significant Central European and Balkan cities [34] with an important role in the political evolution, settlement patterns and urban agglomerations development beyond national borders [35,36]. Yet, the Danube also divided and served as natural border since the Roman Empire. In modern times, the Bulgaria-Romania border was firstly established on the Danube in 1829 (Treaty of Adrianople, concluding on the Russo-Turkish War). As mentioned earlier, the delimitation was actually done in 1908. Since then, no change of the border line was approved. The Danube represents the border between Bulgaria on the right bank and Romania on the left bank, between rkm 845 and rkm 376 (Fig 1a).

The Danube is one of the largest rivers in Europe (the second one after the Volga), but less scientifically studied than the Rhine for example [34]. The Danube forms sectors with different features depending on the landforms they cross, as well as specific ecologic and cultural values [39]. The lower course of the Danube corresponds to the entry in Romania or to the exit from the Iron Gates gorges according to various sources [37]. Along the lower course, the Danube has a discharge of approximately 6,077 m^3^/s at Oltenița (rkm 429.8) with a maximum of 17,303 m^3^/s in April 2006 and a minimum of 1,060 m^3^/s in January 1858 [40]. The high flows usually occur in April due to snow melt, while the low flows in October in lack of precipitation. The Danube recorded major floods in 2005, 2006, and 2010; since then, the Danube did not sufferend high magnitude floods; contrarely, low discharges were recorded during last years (Fig 1b). The mean suspended sediment load reaches 1,368 kg/s (median particle size = 0.025 mm), while the bedload is of only 13.17 kg/s (median particle size = 0.294 mm) [40]. The riverbed is characterized by medium sands [41]. We point out that the Bulgarian bank is higher (more important elevention and slope toward the Danube), while the Romanian bank is lower with a large floodplain (Fig 1c).

Despite fluvial metamorphosis upstream on the upper and middle courses, the Danube generally maintained its pattern on the lower course, including along the Bulgaria-Romania border. The river forms a low-energy multi-thread anabranching channel (Fig 1d). The floodplain channels, lakes and islands disappeared due to diking, desiccation and draining [42]. The main channel continues to form islands [43] that slowly migrates downstream [44]. Yet, sediment excavation triggered bed incision and bank erosion [41]. A riverbed incision of approximately 3–4 m and locally up to 6–9 m was recorded; also, the river channel may move laterally in response to river-bank erosion, as the Danube channel has no systematical bank-reinforcement [41]. Various changes determined the decrease of the suspended sediment load especially after the construction of the two hydropower plants [45] of Iron Gates I (since 1972) and II (since 1986). H. Habersack and collaborators [45] consider that the sediments the Danube transports nowadays come mostly from its banks. We underline that the main drivers of these pressures and alterations of the Danube River multi-thread channel were: the navigation, hydropower generation, and agriculture in the floodplain.

## Methodology

The islands are studied at four time-scales: (i) the century scale shows the main trends of spatial dynamics of islands since the designation of the borderline; (ii) the half of the century scale indicates the islands dynamics under engineering works; (iii) the scale of the last decades is appropriate to understand the potential of changes of the islands in terms of dominant processes under the present-day hydrological conditions, and (iv) the present-day scale shows the reality in the field.

### Data

Data on the channel and islands forms and dimensions were created based on the documents in Table 1. In the case of georeferenced documents in GIS, we delignated the banks of the active channel, including the point bars devoid of vegetation. Then, inside the active channel, we delignated islands covered by vegetation and bars devoid of vegetation. In the case of islands, the water level is not necessarily a limit. The islands are represented as having high banks on the Military Survey Maps. According to fieldwork, present-day islands have over 2 m of height with scarp banks, therefore, they are not exposed to the water level variability, except for extreme events. On the contrary, the bars are dependent on the water level–unknown on some of the documents we used, therefore, the interpretation of their spatial dynamics is open to critics. Additionally, we overlapped the official state border line over the Danube River channel and islands. Besides the official spatial error of 10 m, all interpretations compared to the countries border depend on the limitations and errors of all the other documents that were used. In the case of non-georeferenced documents, we only numbered selected indicators. The interpretations of the islands’ spatial dynamics were validated by the field work. We visited approximately 10% of all the Danube islands on the Bulgaria-Romania border, such as the case studies in Fig 1c.

**Table 1:** Documents used for analyzing the spatial dynamics of the Danube islands.

Document	Date	Spatial scale/resolution	Source	Limits	GIS potentially errors
**GIS**					
Landsat and Sentinel satellite imagery	1984–2022 (selected scenes)	10–30 m	https://earthexplorer.usgs.gov/ https://scihub.copernicus.eu	Bars and point bars depending on the water level. Satellite scenes were selected during low water flows.	Vectorization errors at the border between water and land due to the medium spatial resolution of these scenes (e.g., canopy shadow)
Military Survey Maps	1898–1916	1:20,000	http://igrek.amzp.pl/mapindex.php?cat=ROMLCH020K	No information about the water level during the topographic survey	Georeferencing 80 m root mean square error = (generally toward South-East, estimated based on 50 randomly chosen points located in the main Romanian towns) due to the lack of markers in riparian environment over more than 100 years
State border line	Present-day	-	https://geoportal.ancpi.ro/	Interpretation depending on the previous data	Errors of 10 m estimated by the data provider
**Other support documents**					
Pilot charts	1954, 1968, 1994	1:50,000	Danube Commission	All representations are at fairway depth of 2.5 m or Low Navigable Water Level (ENR)	Without georeferencing
Bulgarian Map of the Danube islands	1908	unknown	National Archives of Bulgaria	Fairway and border	Approach based only on quantifying the rkm
Danube Commission Map	2023	-	https://www.danubecommission.org/dc/en/danube-navigation/danube-ports-map/	Fairway and border	Approach based only on quantifying the rkm

### Indicators

Our paper is based on the matrix of indicators proposed by J.R. Wyrick and P.C. Klingeman [9] that is inspired from other evaluations of the riparian physical habitat (e.g., citations of [46,47]), but it is adapted to the fluvial islands. The matrix includes three main groups with several criteria and classes. Each class is characterized by one or more indicators (especially quantitative, but also qualitative) based on data available in our study. Table 2 provides the classification of J.R. Wyrick and P.C. Klingeman [9] in the first three columns and the methodology proposed in this paper, in the last two columns.

**Table 2:** Revisiting the fluvial island classification by J.R. Wyrick and P.C. Klingeman [9].

Initial scheme			Methodology in this paper		
Group	Criterion	Class	Indicator	Time scale	Spatial scale
Geometric	Location in the thalweg	Near the main thalweg	-Number of channels on cross section every 10 km -Position of the thalweg compared to BG and RO banks -Sinuosity index -Depth of the thalweg -Number of islands -Length of point bars	Century, with focus on the second part of the 20^th^ century	469 km
		Away from the main thread			
	Hydrodynamics shape	Streamlined	-Circularity ratio (C) $C=\frac{4\pi A}{P^{2}}$ -A = area of an island -P = perimeter of an island	Century	469 km
		Angular			
		Irregular			
	Ratio of island width and flow width	Wide (ratio > 1.5)	-Width measurement every 20 km	Century	469 km
		Equal (0.5 < ratio < 1.5)			
		Narrow (ratio < 0.5)			
	Island abundance	None	-Number of islands on cross section every 1 km -Part (%) of the area of the active channel	Century	469 km
		Occasional			
		Frequent			
		Split			
		Braided			
Biophysical	Vegetation recruitment	Mature/Mixed	-Satellite or field observations	Present-day	10% of islands
		Pioneer			
		None			
	Sediment composition	Bedrock	-Field observations	Present-day	10% of islands
		Boulder/Cobble			
		Cobble/Gravel			
		Sand/silt			
	Sediment origin	In channel	-Field observations / documentation	Present-day	10% of islands
		Floodplain			
Inferred	Formation origin	Non-alluvial	-Expert opinion	Century	469 km
		Fluvial			
		Floodplain			
	Island age	Ancient (age > 100 years)	-Expert opinion	Century	469 km
		Mature 15 years < age < 100 years			
		Recent (age < 15 years)			
	Types of changes	Accretion	-Expert opinion and demonstration of case studies	Century, with focus on the last decades	469 km, with case studies
		Erosion			
		Stable			
	Causes of change	Incision in channel	-Expert opinion	Century	469 km
		Aggradation in channel			
		Erosion by floods			
		Flow realignment			
	Potential for change	None	-Expert opinion	Century	469 km
		Stable			
		Changeable			
	Persistence	Long-term	-Expert opinion	Century	469 km
		Intermediate			
		Short-term			

According to the original method, the geometry of islands is characterized by the location in the thalweg of islands, shape, ratio of island width and flow width, and abundance. A special attention was given to the thalweg. The biophysical habitat is characterized by vegetation recruitment, sediment composition, and sediment origin. The inferred characteristics were interpreted from the evolution of islands (formation origin, island age, types of changes, causes of change, potential for change, persistence).

The indicators for geometry were calculated over the last century along the entire Bulgaria-Romania border. The biophysical indicators were estimated at present-day scale by documentation and field work for 10% of the islands that were visited. The inferred indicators derived from the interpretation of the previous results obtained for the entire Bulgaria-Romania border; supplementary analyses were necessary at the time scale of the decade and for a few islands in order to better understand the fluvial processes.

To compare the quantitative fluvial elements, i.e., geometry, and draw statistically demonstrated results, we used the Mann-Whitney test for α = 0.95 and p-value < 0.05. All the comparisons were conducted by XLSTAT.

## Results

Table 3 regroups the main features of the Danube islands on the Bulgaria-Romania border. Further, results are detailed for the evolution during the last century, period of engineering works, and present-day conditions.

**Table 3:** Characterization of the Danube islands on the Bulgaria-Romania border.

Main results			Other explanations (p-value for 1900–2022 or 1954–1994 comparisons, if applicable)
Geometric	Location in the thalweg	Near the main thalweg	The median number of channels on cross-section is 2 (p = 0.081 for 1900–2022). The thalweg migrated toward the Romanian bank (p = 0.030) for 1908–2022. The sinuosity of the thalweg maintained at ~1.2 (1900–2022). The mean channel depth at ENR maintained (1954–6.3 m; 1994–6.4; p = 0.865). The point bars length decreased for 1954–1994 (p = 0.001).
	Hydrodynamics shape	Streamlined	Islands have an elongated form (median C 1900 = 0.381; median C 2022 = 0.359; p = 0.528).
	Ratio of island width and flow width	Narrow	The ratio of island width and flow width was stable at approximately 0.15 (p = 0.589).
	Island abundance	Frequent	The median number of islands on cross section is 1 (p = 0.231). The islands area decreased by 5% out of the total area of the active channel (1900–2022).
Biophysical	Vegetation recruitment	Mature/Mixed Pioneer	Islands are generally forested with patches of natural and planted trees. Some bars were recently colonized by vegetation.
	Sediment composition	Sand and gravel	90% of the islands have an associated bar. Bars are generally formed of sands. A partition of gravels and silts was identified locally on some islands.
	Sediment origin	In channel	According to H. Habersack and collaborators [45], most of the Lower Danube sediment come from bank erosion. A part of the sediment could come from upstream or the tributaries.
		Floodplain	
Inferred	Formation origin	Fluvial	We found no scientific information that islands have another origin but fluvial. According to our investigation, no island formed by avulsion between 1900 and 2022.
	Island age	Ancient Mature Recent	Half of the vegetated islands migrated or merged with each other during the last century while the other half formed during this time interval. Bars are forming/changing every couple of years.
	Types of changes	Accretion Erosion Stable	At the scale of the last century, accretion was dominant; yet, erosion was not neglectable. The number of islands decreased. The area and perimeter increased without statistical significance (for A, p = 0.096; for P, p = 0.080). Under present-day conditions, islands are stable while bars are forming by accretion and reshaping by erosion.
	Causes of change	Aggradation in channel Incision in channel	Pilot charts of 1954–1994 showed the stability of fairway depth. Other publications showed the local incision of the Lower Danube channel during [41]. We believe that the Lower Danube channel’s incision is followed by bank collapse conducting to further aggradation of the channel similar to the processes occurring when excavating sediments from a sandy river [48].
	Potential for change	Stable Changeable	Under present-day conditions, islands are quite stable and bars are changeable.
	Persistence	Long-term Intermediate Short-term	Under present-day conditions, bars are dynamics on short-term.

### Evolution of the channel and islands geometry over the last century

**Fig 2:**
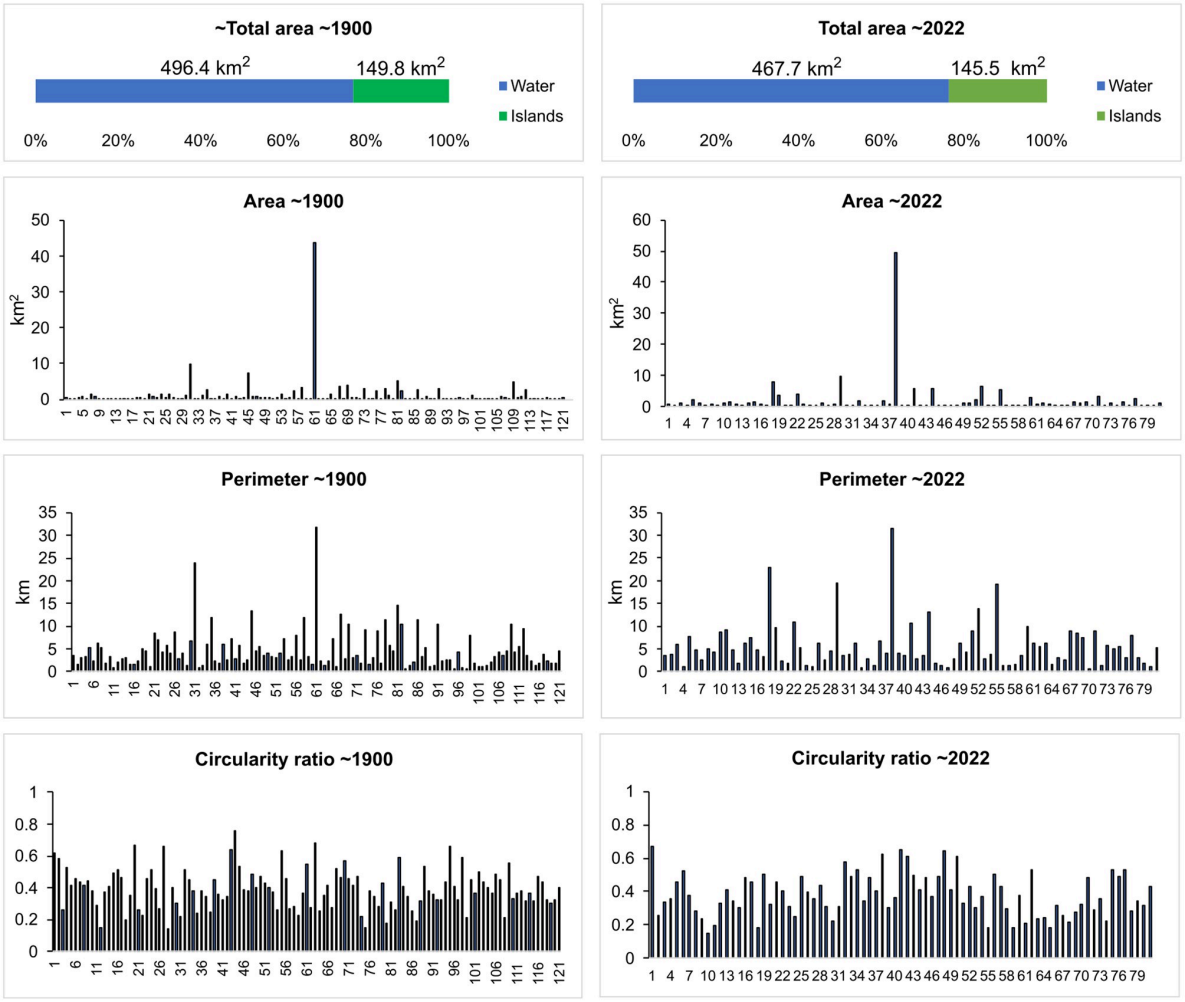
The main features of the Danube River active channel in ~1900 versus ~2022.

The total area of the Danube active channel (including all branches separated by mid-stream alluvial bars and point bars) decreased by 5% during the studied time interval (1900–2022) (Fig 2). The balance of islands out of the total active channel maintained at around 23%. The total area of islands decreased by 2.9%, their number also dropped by approximately 33%, while the median number of islands on cross section remained 1. The mean dimensions of islands in ~1900 were: area of 1.2 km^2^, perimeter of 4.6 km, and circularity ratio of 0.4. The mean dimensions of islands in ~2022 were: area of 1.8 km^2^, perimeter of 5.5 km, and circularity ratio of 0.4. These changes of islands dimensions were not statistically significant.

**Fig 3:**
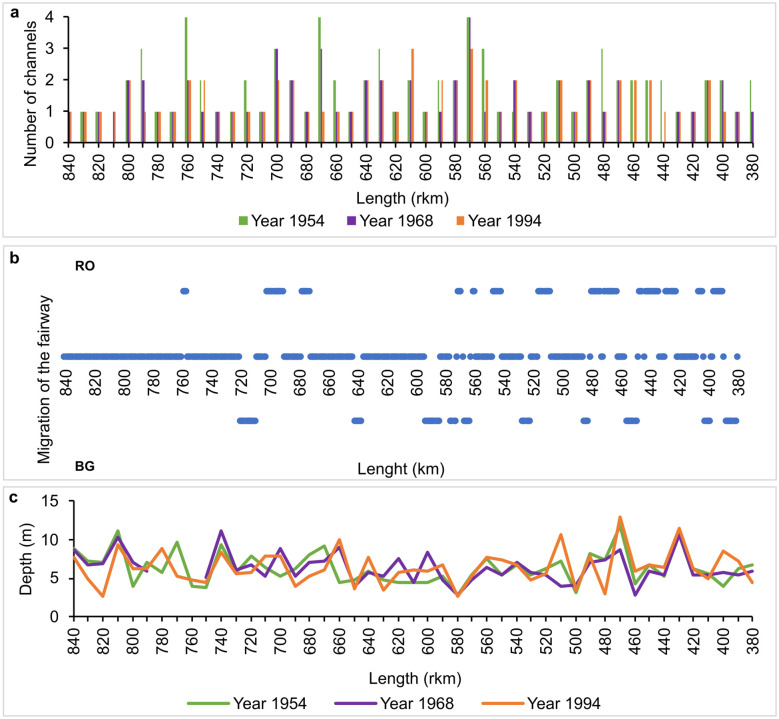
The main features of the Danube River active channel in 1954–1994.

During the last century, the sinuosity of the main channel (fairway) maintained around 1.3. The median number of channels per cross section remained 2. The mean depth of the channel was stable at around 6.4 m (Fig 3). As single statistically significant change, the thalweg migrated toward the Romanian bank between 1900 and 2022 (p = 0.030). The length of point bars changed significantly between 1968 and 1994, with lower values in 1994 (mean value -63% on the Romanian bank, p-value = 0.001; mean value -41.8% on the Bulgarian bank, p-value = 0.021).

### Patterns of inferred processes during the last century

**Fig 4:**
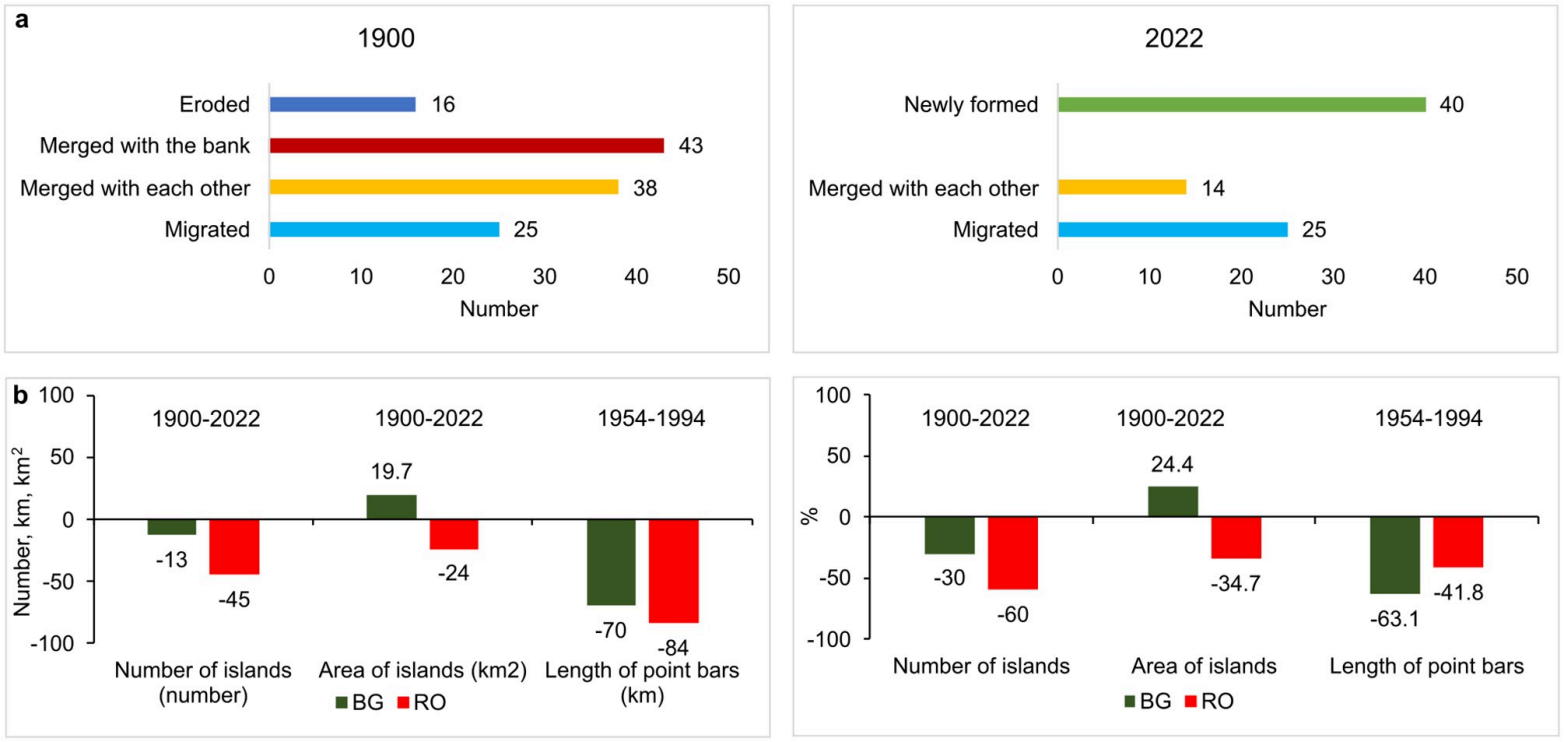
The islands’ spatial dynamics during the last century: a) patterns of evolution; b) comparison to the countries’ border.

At century scale, we found five patterns of evolution of vegetated islands between ~1900 and ~2022 quantified in Fig 4a. (i) A large number of islands merged with the bank (35.2% in ~1900) and were later eroded (approximately half of them). (ii) Other 31.1% of the islands in ~1900 merged with each other; the mean number of merged islands during the studied period was 2.7. (iii) 20.5% of the islands in ~1900 modified their position until ~2022 (e.g., grew and migrated sidewise or downstream). (iv) The balance of 13.1% of islands in ~1900 were eroded during the last century. (v) New islands emerged (approximately half of all islands in ~2022), with unreconfigurable trajectory; they were probably independent bars that were colonized by vegetation during the 20^th^ century. Case studies of islands’ evolution can be found in the sections below.

When compared to the present-day border line, in ~1900, 75 islands (66.9 km^2^ or 44.7% of their total area) were on the Romanian side, while 43 (extended on 72 km^2^ or 48% of their total area) were on the Bulgarian side; 4 islands were on the state border (2.3 km^2^ in Romania and 8.6 km^2^ in Bulgaria or 7.3% of their total area). In ~2022, geographically, 30 islands (36.4 km^2^ or 24.9% of their total area) had major part of their area toward Romania, while 38 toward Bulgaria (91 km^2^ or 62.5% of their total area); 13 islands were on the state border (8.8 km^2^ in Romania and 9.3 km^2^ in Bulgaria or 12.6% of their total area). Fig 4b regroups fluvial adjustments suffered by Bulgaria and Romania in comparison to the countries’ border.

As qualitative approach, we noticed that more islands on the Romanian side merged with the low bank over the last century. They were later eroded. Therefore, we expect to find intense processes of accumulation and erosion on the Romanian bank over the last century. The georeferencing error of the historical maps could be considerable, therefore we do not attempt to estimate the area of bank erosion or accumulation by superposition in GIS.

### Demonstration of inferred processes at recent time scale

**Fig 5:**
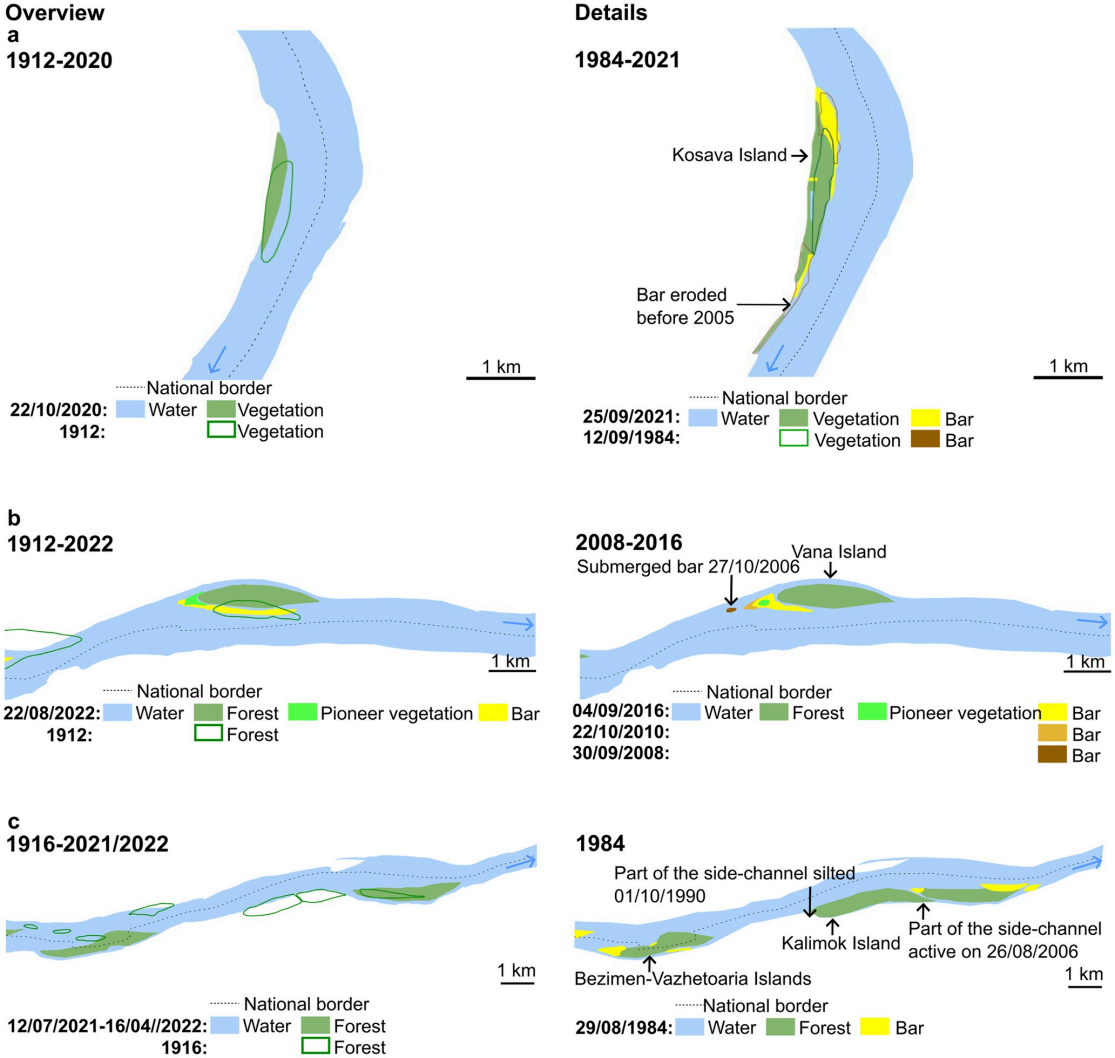
Case study of islands evolution: a) Kosava Island; b) Vana Island; c) Bezimen, Vazhetoaria, and Kalimok, islands; d) Ciocanesti and Denvya islands.

**Fig 6:**
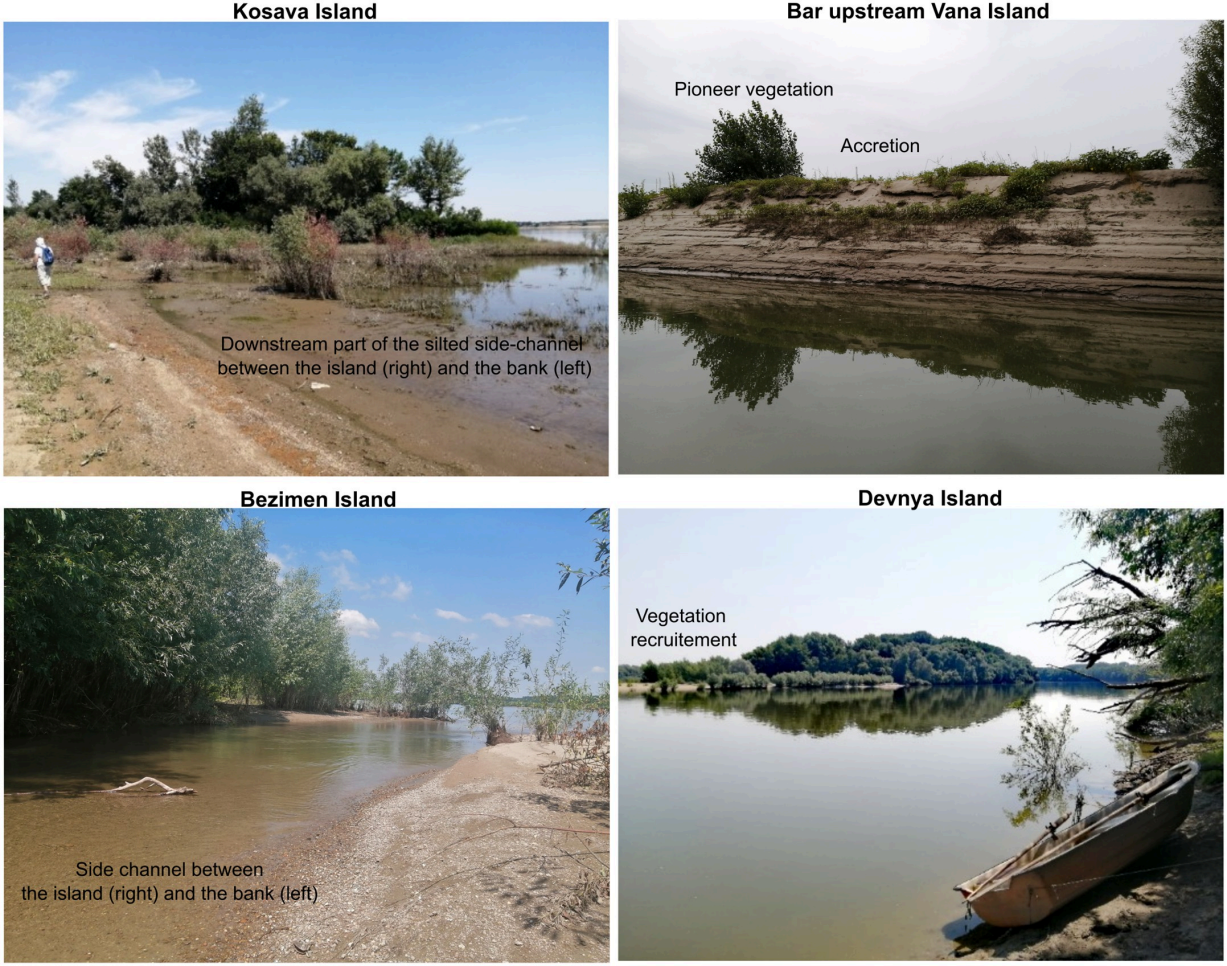
Observations on the Kosava, Vana, Bezimen, and Devnya islands (photos by G. Ioana-Toroimac).

Further, we present some case studies that are relevant for the present-day processes of the Danube islands on the Bulgaria-Romania border (Figs 5 and 6).

At the scale of the last decades, the Kosava Island was relatively stable. The side-channel between the island and the bank silted gradually especially after 1990. In 2021, a small central part of the channel was still covered by water. We predict the merge of the island with the bank.

The Vana Island also was relatively stable. For the bar upstream, the trajectory can be reconstituted. The bar probably formed since the major flood of 2006 as a lee-deposit when a channel obstruction created a downstream zone of shallow depth and reduced velocity. If the river exhibits sufficient sediment load, this wake zone will have reduced transport capability, thus, accumulating sediment and nucleating a fluvial island. The bar became emerged in 2008. Until 2010, it migrated downstream toward the island. Since 2016, its central part became recruited by vegetation. The vegetation extended until 2022, while the sandy bar changed the position and became elongated in accordance to the Danube flow.

The Bezimen-Vazhetoaria islands are a typical example for islands located on the countries border. Several small islands merged with each other and migrated toward the Bulgarian bank during the last century. In 1984, the island was separated from the bank by a small size channel. In 2022, the channel was still functioning.

The Kalimok island formed from two previous islands that merged with each other. In 1984, Kalimok was still an independent island. In 2022, the island was part of the Bulgaria bank. We estimate that the upstream part of the side-channel became silted in 1990, while the downstream part later. We are not able to detect the precise moment when the island merged with the bank due to the low resolution of the satellite imagery. The field work revealed that forest exploitation contributed to the silting of the side-channel; forestry products were deposited in the close vicinity of this small channel.

The Ciocanesti Island formed from two smaller islands that merged with each other. In the last 40 years, the Ciocanesti and Devnya islands became merged by an alluvial bar. Small parts of the alluvial bar were recruited by vegetation. The upstream part of the bar remained active while the downstream part was eroded. The Bulgaria-Romania border crosses the active alluvial bar.

**Fig 7:**
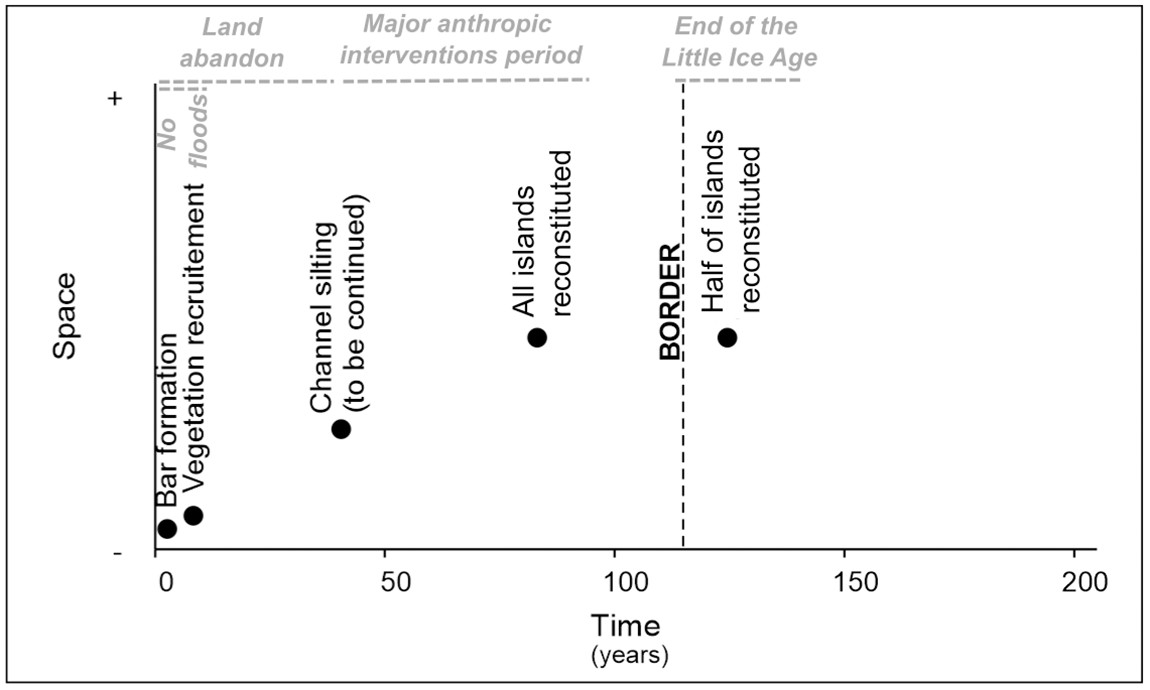
The synthesis of the temporality of islands evolution based mostly on case studies in Fig 5, including the main causes of change.

The temporality of the studied islands evolution is synthesized in Fig 7. In the case of the Vana Island, the bar upstream formed post-2006 flood due to channel aggradation. The bars evolve by accretion during annual high flows. The bars are stabilized by vegetation at a scale of less than 5 years. In the case of Ciocanesti Island, vegetation started to colonize the bar only one year post-2006 flood. In the case of Kosava and Bezimen-Vazhetoaria, the side-channel between the island and the bank is silting for more than 40 years and the merge was not completely achieved. The trajectory of all islands can be reconstituted at a scale of 70 years (since 1954). The trajectory of half of the islands can be reconstituted at the scale of approximately 120 years (since ~1900).

## Discussion

The paper studied the Danube islands on the Bulgaria-Romania border and showed their geometry and evolution in the last century, mostly based on GIS techniques. The Danube has frequent islands, almost 1 island on cross-section every 1 km, streamlined and covered by vegetation. The islands are relatively small (i.e., mean area = 1.2 km^2^) and the majority have an associated alluvial bar. The number of islands decreased over the last century while their total area maintained as well as their share out of the Danube active channel area. Other fluvial elements recorded statistically significant change–the fairway migrated toward the Romanian bank, and the point bars length reduced. We conclude that significant changes were related mostly to the thalweg moving during the studied time interval, while islands maintained their general features.

The islands formation is typical for the fluvial pattern of the Lower Danube River. Going back to 1856, we found an identic fluvial pattern of the main channel, with slightly more extended alluvial bars and less extended vegetated islands [3]. Further, we explain the factors of the evolution of the Lower Danube River channel by period (Fig 7).

- At the end of the Little Ice Age (second part of the 19^th^ century), the presence of fluvial islands with active bars is demonstrative in the Lower Danube Plain in the context of an excess of sediment [49] and high morphogenic floods [50,51].
- In the first part of the 20^th^ century, intense changes probably occurred (i.e., half of the islands changed their position in the study area) in search for a new equilibrium under new hydroclimatic conditions at the end of the Little Ice Age, for which there is not specific topographic or remote survey in Romania [52].
- In the second half of the 20^th^ century (‘major anthropic interventions period’), we expected changes due to a decrease of the sediment load (especially in suspension) post-damming and diking in the study area [49,53]. Hence, the morphology of the Lower Danube River channel was less impacted by this drop of the suspended sediment load. This is explained by the fact that the Lower Danube has the behavior of a sandy river: less sediment load translates by channel erosion, that triggers also lateral erosion of low cohesivity banks according to stream evolution models [54]. The hypothetical bank erosion is confirmed by the significantly decrease of points bars along our study area, yet the continuous formation of in-stream bars. Concerning the significantly statistical change of the thalweg position, it is probably related to the dredging actions to maintain the fairway for navigation under natural constraints (i.e., asymmetric river banks).
- In the last decades, the intensity of formation and migration of islands and bars was less important in the study area. Additionally, bars became recruited by vegetation in our demonstrative case studies (also statistically confirmed by previous works [55,56]). The development of vegetation may be related to land abandonment post-socialism (post-1990) toward rewilding of river environment [57]. The process may be also explained by the decrease of the peak and duration of large floods [58] that do no longer overflow and move emerged landforms. The recent decrease of the intensity of fluvial processes and recruitment by vegetation may be related to the lack of high magnitude recent floods (post-2010) on the Danube [37]. Additionally, the Lower Danube River behave less intense, more naturally and is more stable nowadays due to measures to decrease the sediment extraction for economic purposes or feeding the river with dredged sediment from fairway maintenance for navigation (according to the Danube Commission).
- In the last years, the recent trend of “stability” probably continues as the Lower Danube River recorded long periods with low discharges in summer [59].

To resume, the evolution of the islands along the Lower Danube River continues the general trend recorded by the Danube along the upper and middle sector toward their disappearance and/or stabilization [60,61], but the entire geomorphological process is less intense.

The diachronic study is also a contribution to the fluvial island dynamics and more widely, to the anabranching fluvial systems. Some patterns of the spatial evolution of Danube islands were identified: islands merged with the bank, islands merged with each other, islands migrated, islands were eroded, and new islands formed. Stabilization of the bars by pioneer vegetation was also found. Half of the islands are ancient with a starting point that is not visible on the documents we used. Half of the islands are mature and their trajectory can be reconstituted at least since the middle of the 20th century. The 100-yr flood of 2006 was not necessarily morphogenic and only small-scale adjustments occurred [3]. Nowadays, the islands are relatively stable and only the bars are changeable until being recruited by vegetation, which is similar to other anabranching rivers [62]. In time, this could be translated by islands merging with each other or with the bank by redistribution and storing of the surplus load specific to anabranching rivers under conditions of sediment excess [6]. Hence, this is a slow, predictable process according to Fig 7. This confirms that fluvial islands of low-energy anabranching rivers form slowly, on multi-decadal morphogenesis or even centennial for the largest forms [16]. The Lower Danube River is a good example in Europe for the study of anabranching rivers with low energy and their variety of fluvial processes [55].

The classification scheme of J.R. Wyrick and P.C. Klingeman [9] proved to be a complex tool in the study of islands. Firstly, it contained numerous indicators demonstrative for the islands features and proposed a scale of interpretation of each one of them. Secondly, it gave an overview on both historical conditions and present-day conditions of the islands. Thirdly, the method highlighted the changes in geometry of the active channel over the last century. Finally, it pointed out the main way of evolution of the Danube islands–by lee deposition and stabilization of a bar. The Danube islands along the Bulgaria-Romania border do not evolve by avulsion (fracturing the floodplain), lateral shifts in channel(s) position or by channel incision (isolating certain islands) according to the classification of W.R. Osterkamp [10] further developed within the matrix of J.R. Wyrick and P.C. Klingeman [9]. When compared to other classifications (e.g., [63]), the question of the relation between the geometry and the evolution pattern of the islands remained open due to the lack of documents for the first part of the 20^th^ century and earlier, when the majority of vegetated islands formed.

As limitations of our study, we underline the necessity to observe and understand the overall picture of the Danube islands position, fluvial processes and evolution. Our study is not demonstrative for spatial details. We noted errors related to georeferencing (i.e., 80 m root mean square error due to the low number of common points between old maps and recent satellite imagery), manual vectorization and water level (i.e., the interpretation of the fluvial conditions depends on the last two issues). These errors are inherent when working on historical maps and under water level variations [64]. In future studies for border related decision making, the use of recent satellite imagery (i.e., correct georeferencing, date chosen for mean hydrological conditions) would solve major part of these technical issues.

## Conclusions and recommendation

On the Bulgaria-Romania border line along the Lower Danube River, the spatio-temporal dynamics of the islands from one side to another triggers their declaration as neutral territory and, thus, the exit from use and valorization by local communities. In this context, our paper analyzed the fluvial islands dynamics when compared to the national border. We studied the islands by the diachronic cartography in GIS. We highlight the following findings. The Danube is an anabranching river transporting mostly sands that is self-adjusting toward a transport-efficient geometry of the river channel. The evolution of the Danube vegetated islands is a slow process that may take several decades. The Danube islands follow several patterns of evolution: formation and migration of alluvial bars until merging with an island or until side-channel silting and merging with the bank. In the case of the Lower Danube River, the redistribution of sediment as trigger of a pattern of evolution can occur in a couple of years. The Lower Danube respects its historical background; therefore, bars formation is more likely to occur in the same place as several decades ago. Our demonstration of fluvial islands evolution, despite training structures at basin scale, recommends the Lower Danube River as a good example in Europe for the study of anabranching rivers with low energy.

Consequently, under present-day hydrological conditions, the evolution of the Lower Danube River’s forested islands is not unexpected or spectacular. In this context, we recommend:

1. to continue the monitoring of the islands and bars through remote sensing imagery and automatic techniques and to interpret their geomorphological trajectory in relation to the water level;
2. to continue with the ‘top-down approach’ when concerned the topographic and cadastral decisions related to islands;
3. but to open up to a ‘bottom-up approach’ when concerned the use of stable islands, thus, listen and respond to the necessity of local communities [65].
